# Mast cells activation and high blood tryptase levels due to paclitaxel administration. Is Cremophor EL the culprit? A case report

**DOI:** 10.1097/MD.0000000000022814

**Published:** 2020-10-23

**Authors:** Stefano D’Errico, Benedetta Baldari, Mauro Arcangeli, Alessandro Santurro, Paola Frati, Vittorio Fineschi

**Affiliations:** a Department of Medicine, Surgery and Health, University of Trieste, Trieste; b Department of Anatomical, Histological, Forensic and Orthopaedic Sciences, Sapienza University of Rome, Rome; c Department of Life, Health and Environmental Sciences, University of L’Aquila, L’Aquila, Italy.

**Keywords:** case report, cremophor EL, fatal anaphylactic shock, hypersensitivity reaction, mast cells degranulation, paclitaxel, tryptase

## Abstract

**Rationale:**

Although the cancer incidence continues to rise, cancer mortality has declined over the past decade, in large part due to more efficacious chemotherapeutic regimens thus, the ability to use first-line chemotherapeutic agents in the treatment of patients with cancer is crucial. Antineoplastic agents can potentially cause toxic and/or hypersensitivity reactions, that can have serious consequences. Anaphylaxis is a big pitfall in oncological patients; the most important aspect in diagnosing anaphylaxis is to precisely identify the offending agent to prevent future events. Paclitaxel (Taxol) is widely used as antitumor medication in the ovarian, breast, non-small-cell lung, and other cancers. Paclitaxel hypersensitivity reactions are frequently described in the literature, but fatalities are rarely reported. Due to the low solubility of paclitaxel, the compound requires dissolution in Cremophor EL, a derivative of castor oil.

**Patient concerns:**

A 79-year-old man was affected by high-grade non-papillary urothelial carcinoma and underwent a radical cystectomy and prostatectomy with locoregional lymphadenectomy.

**Diagnosis:**

Eight months later, relapse was detected, and penis amputation and left nephrostomy were performed. Multiple metastases to lymph nodes were detected.

**Interventions:**

Palliative chemotherapy was started with Paclitaxel (110 mg) infused at a rate of 50 mL/h. Despite premedication with cetirizine dihydrochloride, dexamethasone, ondansetron, ranitidine, 20 min after Paclitaxel infusion starts, the patient developed general distress, followed by cardiac arrest.

**Outcomes:**

The mechanism of fatal paclitaxel-associated hypersensitivity reaction is uncertain and its solvent vehicle Cremophor EL may be involved. Several mechanisms have been postulated: an IgE-mediated mast cell degranulation induced by paclitaxel or Cremophor EL, a non-IgE-mediated idiosyncratic mast cell degranulation by paclitaxel or by Cremophor EL, and complement activation. Severe hypersensitivity reactions with fatal outcome are considered rare.

**Lessons:**

The unpredictability and often dramatic reactions of Taxol cause substantial anxiety for doctors and caretakers. They also represent a significant logistic and financial burden on hospitals. Despite premedication, skin testing, and desensitization protocols administration of taxane-based, chemotherapy cannot be considered safe and severe to fatal hypersensitivity reactions cannot be prevented.

## 1 Introduction

Hypersensitivity reactions (HSR) to paclitaxel were reported soon after the beginning of taxol trials in the early 1980s and kept attracting attention.^[1,2]^ The estimated incidence of HSRs after paclitaxel infusion is <44% for mild HSRs and <10% for severe HSRs while the fatal outcome is rare.^[3]^ Symptoms vary from mild pruritus to severe and life-threatening or fatal anaphylactic reactions. HSRs predominantly occur during the first 10 to 15 min of infusion with 78% within the first 10 min, and in 95% of cases reactions occur during the first or second infusion.^[4,5]^ In a few cases, HSRs occur during subsequent infusions. Paclitaxel HSRs range in severity from urticaria, angioedema, erythematous rashes to systemic anaphylaxis with respiratory arrest, cardiac collapse, and death.^[2]^ Some patients developed skin reactions several days or up to a week after infusion. Between 1997 and 2007 in the United States, Europe, and Japan, 171 cases of anaphylaxis caused by cremophor-containing paclitaxel have been reported.^[6]^ In those reports, 58 (34%) of the patients could not survive the anaphylactic shock. In 22% of fatalities, patients received premedication with corticosteroids before the injection of paclitaxel. Medical Research on Adverse Drug Events and Reports (Med-RADAR) also reviewed 6 cases of cremophor-containing paclitaxel-induced anaphylaxis with two fatal cases.^[7]^ A meta-analysis evaluated the impact of infusion time in cases of anaphylaxis produced by paclitaxel and showed no difference in the risk of developing hypersensitivity when treatment was administered over 3 or 24 h (risk ratio: 1.86; 95% confidence interval: 0.63–5.52).^[8]^ Furthermore, Hainsworth et al noted no difference in activity between 1-day and 3-day paclitaxel schedules in which each dose was administered by1-h infusion.^[9]^ The occurrence of HSRs can be influenced by the administration of an appropriate premedication. Paclitaxel-related immediate HSRs occur in up to 30% of patients, with this percentage decreasing to under 10% with the administration of antihistamine and corticosteroid premedication.^[10–13]^ Kwon et al showed that, compared with a single administration 30 min before treatment, administration of dexamethasone 12 and 6 h before infusion of paclitaxel led to fewer HSRs.^[14]^ Other studies showed no difference.^[15]^ Even though, the use of premedication and/or the slowing of infusion rates are effective but not always successful with a small percentage of patients still developing HSRs despite their intense pre-medication with dexamethasone and antihistamines (diphenhydramine, cimetidine, ranitidine).^[16,17]^ Oral premedication with dexamethasone—a long-acting glucocorticoid with a biologic half-life of ∼48 h and noticeable onset of biologic activity after several hours—at a dose of 20 mg (given orally at 12 and 6 h before infusion of paclitaxel) has been shown to reduce the incidence of paclitaxel-induced HSRs significantly.^[18]^ In fact, dexamethasone strongly inhibits inflammation, especially the cellular-mediated immunity and the production or action of the local mediators of inflammation, such as the prostaglandins and lymphokines. Furthermore, dexamethasone reduces vascular permeability and maintains normal vascular responsiveness to circulating vasoconstrictor factors. However, due to logistical factors, short-course premedication with intravenously administered dexamethasone—given 30 min prior to paclitaxel infusion—has become customary in many centers.^[1]^ In an attempt to reduce steroid-induced side effects, especially for patients receiving weekly paclitaxel, dexamethasone dose reduction has been proposed without an increase of severe HSRs.^[19]^

Paclitaxel HSRs are frequently described in the literature, but fatalities are rarely reported. We present a fatal paclitaxel-associated anaphylaxis case in which a complete laboratory workup was performed, with a dosage of the sieric tryptase and immunohistochemical positiveness around the mast cells demonstrating evidence of degranulation to prove trigger activation of mast cells. Tryptase is the most abundant secretory granule-derived serine proteinase contained in mast cells and it is considered a reliable marker for mast cell activation.

## 2 Clinical case

**Figure 1:**
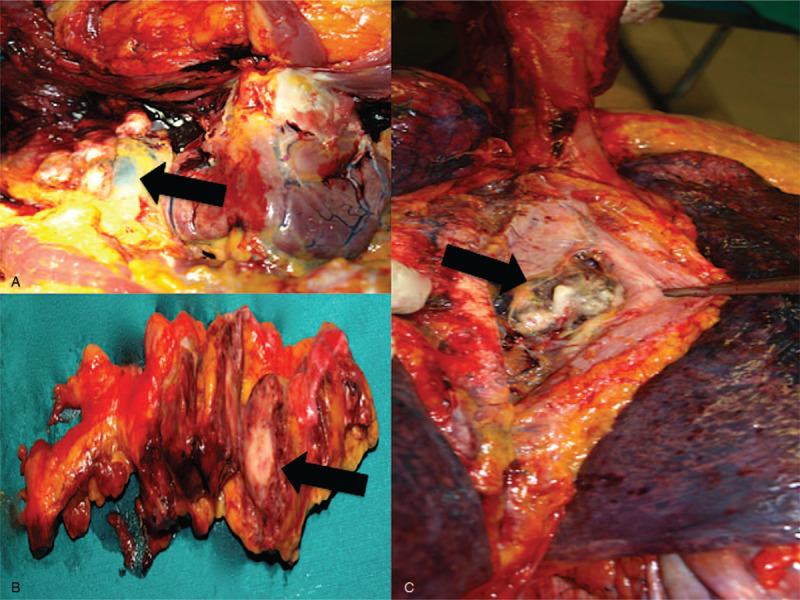
Laterocervical (A), hilar (B), and pancreatic (C) metastases (arrows) of high-grade infiltrating non-papillary urothelial carcinoma treated with radical cystectomy.

**Figure 2:**
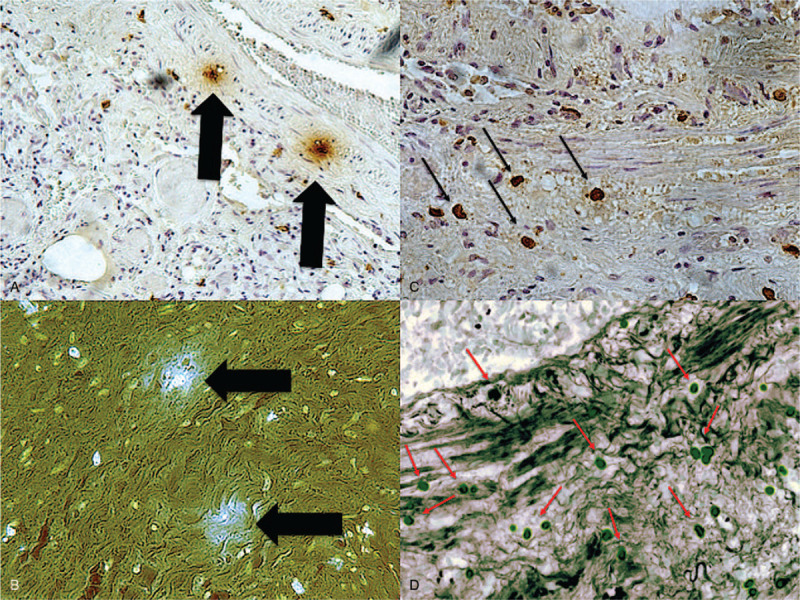
Lung: (A) degranulating mast cells (starry effect in brown, Ab anti-tryptase ×60) with tryptase positive material outside the cells (B) was documented (starry effect in blue, fluorescence ×100). (C) Tryptase positiveness around the mast cells (brown reactions) (Ab anti-tryptase ×40) reveals evidence of mast cells degranulation; an (D) high number of mast cells was documented in the lung (Ab anti-tryptase ×20).

A 79-year-old man was affected by high-grade non-papillary urothelial carcinoma and underwent a radical cystectomy and prostatectomy with locoregional lymphadenectomy. Eight months later, relapse was detected, and penis amputation and left nephrostomy were performed. Multiple metastases to lymph nodes were detected. Palliative chemotherapy was started with Paclitaxel (110 mg) mixed with 500 cm^3^ physiological solution and infused at a rate of 50 mL/h. Despite premedication with cetirizine dihydrochloride, dexamethasone, ondansetron, ranitidine, 20 min after Paclitaxel infusion starts, the patient presented cough and dyspnea. He rapidly developed general distress, followed by cardiac arrest. Paclitaxel infusion was immediately stopped and intramuscular epinephrine (0.5 mg) injection was performed. Cardiopulmonary resuscitation, which included intubation and respiratory support, was started without delay and remained unsuccessful. A complete post-mortem examination was performed two days after death. Autopsy confirming the presence of right ileostomy, radical cystectomy, and left nephrostomy. Additional findings included pulmonary, pancreatic, and adrenal metastasis. Metastatic lymph nodes at pulmonary hilum were also observed (Fig. 1). The heart was increased in size and volume and coronary arteries examination excluded significant obstruction of the lumen. Pulmonary edema was recorded. Spleen and liver were increased in volume, with regular shape. Histological examination revealed mild cerebral edema and acute pulmonary edema mixed to acute pulmonary emphysema. Myocardial interstitial edema was also detected. An immunohistochemical technique was used to estimate the mast cell population, using the anti-tryptase antibody as a mast cell specific marker. Pulmonary mast cells were identified and a great number of degranulating mast cell with tryptase-positive material outside was observed (Fig. 2). Finally, dosing of the tryptases was performed on a blood sample confirming a concentration of 123.0 μg/L (cut-off value of 45 μg/L for tryptase measured post-mortem has been established for anaphylaxis). This case dealt with a death of judicial interest, for which ethical approval and consent were requested and obtained from the Judicial Authority.

## 3 Discussion

The mechanism of paclitaxel-associated anaphylaxis is still uncertain. In fact, most HSRs occur within the first minutes of infusion of paclitaxel, usually after the first or second dose, indicating that prior sensitization is not necessary. For this reason, the main role of IgE in the mechanism of anaphylaxis after infusion of paclitaxel was excluded even if recently a case of IgE-mediated reaction to paclitaxel was proved by skin and in vitro testing.^[20,21]^ Macrogolglycerol ricinoleate has also been implicated in anaphylactic reactions on the basis that it can induce complement activation, giving rise to anaphylatoxins that trigger mast cells and basophils for a secretory response.^[22,23]^

There is substantial evidence suggesting that Cremophor EL (CrEL) may be involved in anaphylaxis induced by paclitaxel.^[24,25]^ Cremophor (CrEL) is a non-ionic emulsifier used to improve the solubility of various poorly water soluble drugs, including paclitaxel consisting of amphiphilic molecules mixture that form micelles in the water acting as a trigger in complement activation.^[26]^ In contrast to earlier reports, CrEL is not an inert vehicle but exerts a range of biological effects, some of which have important clinical implications. The pharmacokinetic behavior of CrEL is dose-independent, although its clearance is highly influenced by the duration of the infusion. This is particularly important since CrEL can affect the disposition of various drugs by changing the unbound drug concentration through micellar encapsulation. Micelles are multimolecular aggregates that usually appear as spherical “core-shell” structures with a dense nucleus surrounded by a less electrodense halo and represent a particulate substance unprotected by surface-bound C regulatory proteins; therefore, satisfying two basic conditions for becoming a C activator. Symptoms induced by cremophor are more likely to be type I HSRs.^[27]^ Activation of complement due to the binding of C3 to their hydrophilic adhesive surface in the absence of immune complex leads to the release of C3a, C5a, and C5b-9, which trigger activation of mast cells, basophils, and other cells via their specific receptors.^[28–30]^ In this case, degranulating mast cells with tryptase positive material outside the cells was documented. Tryptase is the most abundant secretory granule-derived serine proteinase contained in mast cells and it is considered a reliable marker for mast cell activation. Tryptase positiveness around the mast cells reveals evidence of degranulation to demonstrate trigger activation of mast cells.

The concept that C activation by CrEL would underlie HSRs to paclitaxel was based on the demonstration that CrEL fully accounted for C activation by paclitaxel in vitro.^[23]^ Interestingly, there is substantial inter-individual variation, and sensitivity to a specific liposome does not necessarily imply sensitivity to others. HSRs where the allergen can activate C have been tentatively named C activation-related pseudoallergy (CARPA). Symptoms of CARPA are the same as seen in common allergy or classical type I reactions, while others are unique to C activation. Perhaps the most important distinguishing feature of CARPA is the lack of presensitization and reinforcement; the reaction arises at the first exposure to the drug and then it decreases, rather than increases upon repeated exposure. According to Irizarry et al, allergic reactions following the first dose of cremophor-containing drugs can be explained by the wide use of cremophor, which can result in prior sensitization to cremophor.^[7]^ Differently, authors have shown that Cremophor used without paclitaxel and without premedication does not trigger HSRs while paclitaxel alone induced histamine release in 3 healthy controls but cremophor alone did not.^[31]^ It has been observed that similar HSRs have not been reported with a Cremophor-free formulation of paclitaxel that received FDA approval in 2005. Polysorbate 80 was adopted instead of cremophor. Nevertheless, HSRs with polysorbate 80-containing docetaxel have been reported as well.^[24,32]^

Post-mortem dosing of mast cell tryptase is widely performed in forensic practice for the assessment of cases of suspected anaphylaxis.^[33–35]^ However, despite the extensive use of the method, the interpretation of the results is complex in relation to the presence of confounding factors including the state of conservation of the biological matrix and the increase in the levels of tryptase in other conditions different from anaphylaxis.^[36,37]^

## 4 Conclusion

Despite premedication, skin testing, and desensitization protocols administration of taxane-based chemotherapy cannot be considered completely safe and severe to fatal HSRs cannot be prevented. The use of premedication and/or the slowing of infusion rates are effective but not always successful with a small percentage of patients still developing HSRs despite their intense pre-medication with dexamethasone and antihistamines (diphenhydramine, cimetidine, ranitidine). However, due to logistical factors, short-course premedication with intravenously administered dexamethasone—given 30 min prior to paclitaxel infusion—has become customary in many centers.^[1]^

## Author contributions

**Conceptualization:** Stefano D’Errico.

**Investigation:** Benedetta Baldari.

**Methodology:** Paola Frati, Vittorio Fineschi.

**Supervision:** Paola Frati, Vittorio Fineschi.

**Writing – original draft:** Stefano D’Errico, Benedetta Baldari.

**Writing – review & editing:** Mauro Arcangeli, Alessandro Santurro.
